# Reuse of Textile Waste to Production of the Fibrous Antibacterial Membrane with Filtration Potential

**DOI:** 10.3390/nano12010050

**Published:** 2021-12-24

**Authors:** Alena Opálková Šišková, Pavel Pleva, Jakub Hrůza, Jaroslava Frajová, Jana Sedlaříková, Petra Peer, Angela Kleinová, Magda Janalíková

**Affiliations:** 1 Institute of Materials and Machine Mechanics, Slovak Academy of Sciences, Dúbravská Cesta 9, 845 13 Bratislava, Slovakia; 2 Polymer Institute of Slovak Academy of Sciences, Dúbravská Cesta 9, 845 41 Bratislava, Slovakia; angela.kleinova@savba.sk; 3 Department of Environmental Protection Engineering, Faculty of Technology, Tomas Bata University in Zlin, Vavreckova 275, 760 01 Zlin, Czech Republic; peer@utb.cz (P.P.); mjanalikova@utb.cz (M.J.); 4 Institute for Nanomaterials, Advanced Technologies and Innovation, Technical University of Liberec, Studentská 1402/2, 461 17 Liberec, Czech Republic; jakubhruza1@seznam.cz; 5 Faculty of Arts and Architecture, Technical University of Liberec, Studentská 1402/2, 460 01 Liberec, Czech Republic; jaroslava.frajova@tul.cz; 6 Department of Fat, Surfactant and Cosmetics Technology, Faculty of Technology, Tomas Bata University in Zlin, Vavreckova 275, 760 01 Zlin, Czech Republic; sedlarikova@utb.cz

**Keywords:** electrospinning, recycling of textile waste, stocking, polyamide, filtration, antibacterial membrane

## Abstract

Wasted synthetic fabrics are a type of textile waste source; the reuse of them brings environmental protection and turns waste into a valuable material. In this work, the used nylon (polyamide) stockings were transmuted into a fine fibrous membrane via an electrospinning process. In addition, the safety antibacterial agent, monoacylglycerol (MAG), was incorporated into a recycled fibrous membrane. The results revealed that the neat, recycled polyamide (rPA) fibers with a hydrophobic surface could be converted into hydrophilic fibers by blending various amounts of MAG with rPA solution prior to electrospinning. The filtration efficiency and air/water vapor permeability of the two types of produced membranes, neat rPA, and rPA/MAG, were tested. Their filtration efficiency (E_100_) was more than 92% and 96%, respectively. The membranes were classified according to Standard EN1822, and therefore, the membranes rPA and rPA/MAG were assigned to the classes E10 and E11, respectively. The air permeability was not affected by the addition of MAG, and water vapor permeability was slightly enhanced. Based on the obtained data, prepared rPA/MAG fibrous membranes can be evaluated as antifouling against both tested bacterial strains and antimicrobial against *S. aureus*.

## 1. Introduction

Following the current global pandemic situation and prognosis of virology experts, there is no doubt that fibrous membranes as filtration media against COVID-19 are currently one of the most demanded products ever [1]. With this respect, every alternative source of materials, as well as the method of filtration membrane fabrication, should be seriously considered.

Waste is perceived as a significant source of material. Primarily, textile production generates a considerable amount of waste annually due to the growth of the world population and improved living standards. Due to the fast fashion cycle, various wastes and an enormous amount of synthetic textiles are removed every year. According to the literature, 92 million tons of global fashion went to waste in 2015, and only about 15% of it was recycled. An increase in textile waste to 148 million tons is expected in 2030 [2]. Textile waste ends up in either landfills or incinerators. The landfilled textiles in the form of fibers or yarns creates an environmental burden. All types of synthetic fibers, including polyester, acrylic, olefin, and polyamides, have been found in oceans, rivers, and even water treatment plants [3,4]. When incinerated, they create severe environmental issues due to the release of toxic gases. Such environmental issues occur mainly in developing countries, where manufacturing facilities for the developed world are located [5]. In contrast, reusing or recycling aligns well with the circular economy concept, where the textiles are recirculated by extending the product life beyond one cycle.

Textile recycling includes processes on the level of fibers, polymers, and monomers. The suitability of garments for recycling is determined in large part by fiber composition and the chemical structure of the polymers that make up the fibers [6]. The classification of textile recycling and recycling methods, products, and drawbacks is summarized in Table 1.

According to the studies [7,8,9], the first step of recycling mechanical, physical and chemical fibers is cutting, tearing, grinding, and shredding into the individual fibers.

Then in the case of mechanical recycling, nonwoven textiles are produced. Nonwoven materials may be employed for thermal and acoustic insulation applications in automotive or geotextile industries. Fibers can be utilized as reinforcements for construction composites, asphalt, and concrete as well. The mechanical recycling method can be applied for natural, synthetic or mixed natural/synthetic fibers [9,10,11].

The recovered fibers are mainly melted, extruded to pellets, and turned into fibers again by physical recycling into the form of fibers. This method can be applied to synthetic and natural fibers [6,7,13]. Widely used worldwide fleece garments are among the best representative products commercially available from recycled PET. The preparation of fibers from recycled PET to fabricate trendy fleece garments, such as sweaters and jackets, is one of the best examples of proper waste management based on upcycling [14]. The PET waste is melted and re-extruded to realize pellets or filaments and is re-worked into fabric manufacturing processes. An example of the physical recycling method of cellulose fibers is the production of viscose rayon from solution spinning [15].

Chemical/biological recycling includes pyrolysis, enzymatic hydrolysis, hydrothermal ammonolysis, and glycolysis. Chemical/biological recycling can be applied mainly to natural and synthetic fibers. Chemical recycling is carried out by depolymerization into the individual monomer constituents [2,16]. However, enzymatic recycling could be used for natural fibers. The resultants of cotton enzymatic hydrolysis are oligosaccharides, cellobiose, glucose, etc. [12,17]. These products are further used. Bio-ethanol is produced from glucose by fermentation. Hydrolysate can be used as a carbon source in growth media to produce bacterial cellulose [18].

The blended fibers seem to be a challenge. For example, there are two possibilities for separating the individual components in the case of a cotton/polyester blend: (i) cotton is soluble in hydrochloric acid under hydrothermal conditions, and polyester is recovered as such by filtration, and (ii) cotton and polyester are separated by dissolving polyester by an appropriate solvent, e.g., N, N-dimethyl-cyclohexylamine, and cotton was separated by filtration in [19].

Polyamides (PAs) are versatile semicrystalline polymers with high chemical resistance and good thermal and mechanical properties [20]. They are produced from fossil resources, usually in the form of fibers, for use in a broad scope of applications (fashion, automotive, electrical, electronic, construction, packaging, coatings, and other industries). Currently, PAs are produced on an annual worldwide multimillion-ton scale, and the production is estimated to be continuously growing [1]. Therefore, polyamides represent an important proportion of polymer waste.

PA waste is mostly recycled chemically or physically. In the first case, polyamide undergoes ammonolysis, hydrolysis, and alkaline hydrolysis, to achieve monomers of caprolactam or hexamethylenediamine acid (depending on whether the waste is polyamide 6 or polyamide 6.6) and adipic acid. The monomers are used for synthesis. In the second case, the PA waste is melted and re-extruded to realize pellets or filaments and re-worked into fabric manufacturing processes; this is similar to PET recycling [14].

As with the physical method, electrospinning offers the opportunity to prepare fibrous polymer products from virgin and post-industrial or post-consumer polymers (plastic wastes). The randomly placed ultrafine fibers in the electrospun membranes are attractive as filtration materials. The high surface area to volume ratios, nano-porosity, good mechanical properties, and vapor permeability of such nanofibrous membranes predestined them for air, water, or even personal protection against very fine dirt, bacteria, viruses with dimensions smaller than 100 nm, or volatile organic compounds [21,22,23]. The comparison of the mentioned spinning methods is in Table 2.

Recent studies have demonstrated the possibility of turning plastic waste into submicron fibers using the electrospinning technique [25,26,27]. Electrospinning seems to be an easy and cheap technique used to fabricate nanocomposite textile with a highly porous structure suitable for a wide variety of applications. For example, poly(ethylene terephthalate)-based waste drinking bottles were used to fabricate electrospun fibers with an average diameter of 95 ± 37 nm for filtration applications [21], while Isik et al. [24] obtained expanded polystyrene (EPS) from insulation and packing material without further purification. Polymer submicron fibers with hydrophobic and oleophilic characters were successfully electrospun and used as adsorbents for oily water mixtures.

It was already published that the filtration efficiency of electrospun membranes could be high, even very close to 100%. However, the efficiency depends on the basis weight of the electrospun nonwoven textile, the diameter of fibers in the membrane, and porosity [30]. Moreover, pressure drop, quality factor, and air or water vapor permeability are significant parameters for assessing user comfort in personal protection applications [31]. Therefore, it is always challenging to increase efficiency while maintaining low-pressure drops.

Several groups studied electrospun nanofibers with various morphologies by using polymer materials, such as polyacrylonitrile [32], polystyrene [33], poly (ε-caprolactone) [34], cellulose acetate (CA) [35], silk fibroin (SF) [36], poly(lactic acid) (PLA) [37], poly(vinyl alcohol) (PVA) [38,39], polyvinylidene fluoride (PVDF) [40], poly(ethylene terephthalate) (PET) [21] and poly(methyl methacrylate) [41], for use in air filtration applications.

However, there is no systematic study about recycling textile waste by electrospinning. At the same time, polyamide can be easily electrospun within the optimal process/material parameters [42,43,44]. The functionalization of fibers from recycled polymers can improve the mechanical properties or even add new ones, e.g., antibacterial activity or antifouling ability.

According to the authors’ best knowledge, the fabrication of electrospun fibrous membrane from polyamide waste loaded with monoacylglycerols or other surfactants is scarce. Non-ionic monoacylglycerol was selected for its advantageous properties, including high compatibility with other constituents, regardless of the ionic character and solution properties, such as pH, ionic strength, and water hardness. Monoacylglycerols possess antimicrobial activity, depending on the given molecular structure, i.e., the type and the length of a fatty acid carbon chain. They interfere with bacterial cell membranes and might cause cell lysis or a range of indirect effects, inhibiting cell metabolism [45]. Due to the safety of monoacylglycerols, they are commonly utilized in the cosmetic and the pharmaceutical industries [46,47,48].

In this work, the conventional post-consumer women’s stockings made of polyamide (nylon) was used to fabricate electrospun recycled polyamide enriched by monoacylglycerol (rPA/MAG) fibrous self-supporting membrane, and its potential application in filtration was investigated. The motivation for such a study is the demand to develop new materials suitable for personal protection in the ongoing global pandemic situation.

## 2. Materials and Methods

### 2.1. Materials, Chemicals, and Microorganisms

Polyamide (PA) was obtained from used nylon stockings. The PA’s molar mass and the molar mass dispersity were assessed to understand the input material. The molar mass was estimated by gel permeation chromatography (GPC) using trifluoroethanol (TFE) (with purity ≥98%, Sigma-Aldrich, St. Luis, MO, USA) as an eluent with the addition of 0.1 M potassium trifluoroacetate (with purity ≥98%, Sigma-Aldrich, St. Luis, MO, USA) to increase the ionic strength. The GPC system consists of a Shimadzu LC-20 pump (Shimadzu Corporation, Kyoto, Japan), and a Shimadzu refractive index detector (Shimadzu USA Manufacturer Inc., Canby, OR, USA). The two PSS GRAM 5 µm columns (d = 8 mm, l = 300 mm; 100 + 1000 Å) (Polymer Standard Services, Mainz, Germany) at 25 °C were used. Poly(methyl methacrylate) standards (Polymer Standard Services, Mainz, Germany) were used for calibration. The molar mass of input PA was M_w_ = 71,000 g.mol^−1,^ and the molar mass dispersity was Ð_M_ *=* 2.03.

1,1,1,3,3,3-Hexa fluoro-2-propanol (HFIP, >99.0% purity) was purchased from TCI Tokyo Kasei, Tokyo, Japan. Dichloromethane p.a. (DCM, 99.8% purity) was supplied by Lach-Ner, Bratislava, Slovakia. Lauric acid, glycidol, and chromium acetate hydroxide were supplied by Sigma-Aldrich (St. Louis, MO, USA). All the chemicals were applied as received without further purification. Both bacterial strains, *Escherichia coli* ATCC 25922 (Gram-negative rods) and *Staphylococcus aureus* ATCC 25923 (Gram-positive cocci), were obtained from the Czech Collection of Microorganisms (CCM, Brno, Czech Republic). Di-ethylhexyl-sebacate (DEHS, >97.0% purity) was purchased from Palas GmbH, Karlsruhe, Germany.

### 2.2. Preparation of MAG

Monoacylglycerol (MAG) was prepared by direct addition of dodecanoic acid to oxirane-2-ylmethanol by the epoxide ring opening. The reaction was performed in a double skin reactor at the temperature of 90 °C [49]. The product was then recrystallized from ethanol to the purity of ≥98% (according to the ^1^H NMR spectrum). The NMR spectra (see Supplementary Materials, Figures S1 and S2) matched the considered structure and previously published data [50].

^1^H NMR: *δ* 0.86 (t, 3H, ^3^*J*_H,H_ = 6.4 Hz), 1.24 (um, 16H), 1.51 (um, 2H), 2.28 (t, 2H, ^3^*J*_H,H_ = 7.2 Hz), 3.34 (um, 2H), 3.63 (um, 1H), 3.90 (dd, 1H, ^3^*J*_H,H_ = 6.5 Hz, ^2^*J*_H,H_ = 11.0 Hz), 4.03 (dd, 1H, ^3^*J*_H,H_ = 4.2 Hz, ^2^*J*_H,H_ = 11.1 Hz), 4.58 (t, 1H, ^3^*J*_H,H_ = 5.7 Hz), 4.81 (d, 1H, ^3^*J*_H,H_ = 5.0 Hz) ppm. ^13^C NMR: *δ* 14.8, 23.0, 25.3, 29.3, 29.6, 29.8, 29.9, 32.2, 34.4, 63.5, 66.4, 70.2, 173.8 ppm.

### 2.3. Preparation of Fibrous Membranes

Electrospinning was carried out to produce the fibrous membranes from rPA dissolved in a mixture of 1,1,1,3,3,3-hexafluoro-2-propanol/dichloromethane at a concentration of 15 wt% with different amounts of MAG (from 1 to 3 wt%). Electrospinning was carried out under laboratory temperature and humidity (22 °C ± 1 °C and 57% ± 1%) in a horizontal spinning configuration with a flat-end needle with a 0.8 mm (21G) inner diameter. The working tip-to-collector distance was 12 cm. The applied voltage was 20 kV with positive polarity. The voltage was driven by a high voltage power supply (Spellman SL-150W, Bochum, Germany). The solutions were fed by a single syringe pump model NE-1000 (New Era Pump Systems, Inc., Farmingdale, NY, USA). The feeding rate was 0.2 mL·h^−1^. The electrospun fibers were collected on a grounded flat stationary collector coated with aluminum foil.

### 2.4. Characterization of the Fibrous Membranes

NMR spectra were recorded using a Jeol JNM-ECZ400R/S3 (JEOL, Tokyo, Japan) spectrometer operating at 399.78 MHz (1H) and 100.53 MHz (13C) frequencies. ^1^H- and ^13^C-NMR chemical shifts were referenced to the signal of the solvent (^1^H: *δ* (residual DMSO-*d*_5_) = 2.50 ppm; ^13^C: *δ* (DMSO-*d*_6_) = 40.45 ppm). Signal multiplicity is indicated by “d” for doublet, “t” for triplet, and “um” for unresolved multiplet.

After coating a gold layer via a sputtering system, the surface morphology of the fibers was investigated by scanning electron microscopy VEGA 3 (SEM, Tescan, Brno, Czech Republic). The mean diameters of the fibers were determined with the aid of Adobe Creative Suite software (CS5, Adobe Systems Inc., San Jose, CA, USA), wherein 300 fibers underwent analysis from 3 different images. Pore size distribution was determined by image analysis of SEM images (Adobe Creative Suite software); the mean pore size was calculated from over 100 values.

The wettability of the fibrous layers was measured by the sessile drop method at ambient temperature on a Theta optical tensiometer (Biolin Scientific, Sweden) in combination with OneAttension software. Distilled water was applied as the reference liquid, the droplet volume equaling 3 µL. The results are expressed as the average of five measurements.

Nicolet 8700 spectrophotometer (Thermo Fisher Scientific, Madison, WI, USA) was used for ATR-FTIR (attenuated total reflectance-Fourier transform infrared spectroscopy) spectra recording. The spectrophotometer was equipped with a deuterated triglycine sulfate and thermoelectrically cooled (DTGS TEC) detector. The spectra were recorded in the range of 600–4000 cm^−1^ with 4 cm^−1^, using the absorbance mode.

A Linseis combined thermal analyzer L75/L81/2000 (Linseis Messgeraete GmbH, Selb, Germany) was used for thermogravimetric measurements. Approximately 20 mg of the investigated samples were loosely filled into a smaller cylindrical crucible (height: 14.0 mm, diameter: 6 mm) of TG. The analyses were carried out in the nitrogen atmosphere with a flow of 12 L.h^−1^. The temperature was increased from 24 °C up to 500 °C with the heating and cooling rate of 10 °C·min^−1^.

The filtration effectivity (E) was measured precisely on instrument MFP 1000 HEPA (Palas GmbH, Karlsruhe, Germany) according to the demands of the standard EN 1822 applied to high- and very high efficiency air filters with ultra-low penetration (EPA, HEPA, and ULPA) used in the field of ventilation and air conditioning, as well as in technological processes, such as clean-room technology or the pharmaceutical industry. DEHS fluid suitable for producing steady aerosols was used as testing particles, with the particle size of 120–2460 nm. The face velocity was 5.0 cm.s^−1^ and total volume flow was 30 L.min^−1^. The measurements were carried out on three independently electrospun membranes. The quality factor (Q*_f_*) judges the relative overall performance of different membranes calculated from the measurement of filtration efficiency (E) and drop pressure (ΔP). It is defined as in Equation (1) [51].

(1) $$Q_{f}=\frac{-\ln\left(1-E\right)}{\Delta P}$$

Q*_f_* is fairly independent of basis weight [52].

For the measurement of air permeability (B), the FX3300 air permeability tester III (Artec Testnology, Hertogenbosch, the Netherlands) was used. The measurement pressure was set to 100 Pa, and the test sample’s dimension was 20 × 20 cm. The results were evaluated according to EN ISO 9237.

The relative water vapor permeability was measured using the PERMETEST Sensora Skin Model (Sensora, Liberec, Czech Republic) apparatus [53,54]. The device provides measurements required in the ISO Standard 11092. The measurements were carried out at laboratory temperature (20–22 °C), and the laboratory water vapor concentration (humidity) of the parallel airflow of 45%–60% was applied. The samples with dimensions 12 × 12 cm were used.

The air and relative water vapor permeability was measured for the three independently prepared samples.

### 2.5. Antibacterial Activity

(1) The antibacterial activity of neat rPA or rPA/MAG fibrous membranes was tested by the standard agar diffusion technique [55]. The fibrous layers were cut into circular disks (diameter 9 mm). They were placed on Mueller Hinton Agar (Himedia Laboratories Pvt. Ltd., Mumbai, India) plates inoculated with 1 mL of 0.5 McF turbid bacterial suspension (*Escherichia coli*, *Staphylococcus aureus*) in a sterile saline solution. The plates were incubated at 37 °C for 24 h, and the inhibition zones and growth under the samples were evaluated. All experiments were repeated three times.
(2) The growth kinetics of bacterial species were studied using a Tecan microplate reader (M200Pro, Tecan, Männedorf, Switzerland) to examine the fibrous disks (9 mm) containing various concentrations of MAG. The microplate wells were filled with 250 µL Mueller Hinton Broth (Himedia Laboratories Pvt. Ltd., Mumbai, India), 5 µL 0.5 McF turbid bacterial inoculum (rPA fibers with MAG (0, 1, 2, and 3 wt%) or without (control bacterial growth)) and incubated (with shaking) at 37 °C for 24 h. The absorbance values (in nine rounds) were read as optical density (OD600nm) every 30 min. The modified Gompertz equation was used to describe the lag phase of bacterial growth to evaluate the antimicrobial effect of rPA/MAG fibrous membranes [56,57]. A non-linear regression analysis (Marquardt–Levenburgova method) was used for the calculation of the parameters μmax, λ and A for the following conditions: μ > 0, λ > 0 and A > 0. The maximum specific growth rate (μmax) and asymptotic value are given by (Equation (2))
  (2) $$y=A\cdot exp\left\{-exp\left[\frac{\mu_{\max}\cdot e}{A}\left(\lambda -t\right)+1\right]\right\}$$
  where μ_max_ is the maximum specific growth rate (log CFU.l^−1^.h^−1^); λ is the lag phase (h); and A is the asymptote defined as the maximum value of relative microorganism counts (log CFU.l^−1^).

### 2.6. Biofilm Formation Test

The ability of bacterial attachment and biofilm formation on the surface of the rPA and rPA/MAG fibrous membranes were studied by two microscopy techniques on the circular samples (9 mm disks) cultivated in BHI broth (Himedia Laboratories Pvt. Ltd., Mumbai, India) supplemented by 5% *v*/*v* sucrose (Merck, Darmstadt, Germany) with *Escherichia coli* or *Staphylococcus aureus.* The cultivation at 37 °C lasted for 72 h; the samples were prepared for both microscopy techniques.

(1) The samples were washed with sterile saline solution after cultivation and put on sliding glass. They were dyed by fluorescence dye (SYTO^®^9 and propidium iodide) for 10 s and then covered with a square coverslip. Fluorescence microscopy was performed using a fluorescence microscope Olympus BX53 (Olympus, Tokyo, Japan), equipped with Microscope Digital Camera DP73 (Olympus, Tokyo, Japan) and the cell Sens Standard V1.18 (Olympus, Tokyo, Japan) software. The analysis was carried out on a minimum of 20 positions in three replicates. LIVE/DEAD™ BacLight™ Bacterial Viability Kit (Thermo Fischer, USA), based on the protocol [58], was carried out using slight modifications. SYTO^®^9 dyed plasma membranes of all bacteria, while propidium iodide can color DNA of only dead cells. The excitation/emission maxima for these dyes are about 480/500 nm for SYTO 9 stain and 490/635 nm for propidium iodide. Thus, bacteria with intact cell membranes stain fluorescent green, whereas bacteria with damaged membranes (dead) stain fluorescent red.
(2) The sample disks were washed after cultivation by sterile saline solution and left dried at 40 °C. SEM microscopy was then performed as it was described earlier (2.4).

### 2.7. Statistical Analysis

Data were expressed as mean ± standard deviation (SD). Statistical analysis was carried out by one-way analysis of variance (ANOVA) test using Statistica software (version 10, StatSoft, Inc., Tulsa, OK, USA) and OriginPro (version 9, OriginLab, Northampton, MA, USA) at the significance level of *p* < 0.05.

## 3. Results and Discussion

Basically, dissolving PAs is difficult due to two reasons: (a) polyamides are highly crystalline, and (b) solvents for polyamides are believed to act by strong, particular polar forces [59]. Solution processing of PAs is quite challenging since only a few solvents, such as formic acid (FA), acidic acid (AA), cresol, or fluoric solvents, can dissolve them. All of the solvents used to dissolve PAs have severe environmental challenges. A few attempts were made to replace the existing solvents or propose new solvents. Jabbari et al. used the mixture of FA and urea, calcium chloride, and water [59]. Charlet et al. [60] studied the crystallization and dissolution behavior of polyamide 6-water systems under pressure. Papadopoulou et al. [61] mixed FA with trifluoroacetic acid and acetone. Chang, et al. [62] used N-methyl-2-pyrrolidinone (NMP) and N,N-dimethylacetamide (DMAc) for soluble aromatic polyamides. However, according to the best authors’ knowledge, no environmentally friendly acceptable polyamides solvent was used for electrospinning. The alternative to avoiding the solvents completely could be to use melt-electrospinning. However, this technique suffers from difficulties in melting [28].

Mixtures of existing solvents, such as FA and AA with chloroform, acetone, N, N-dimethylformamide, dichloromethane, or even hexafluoro-2-propanol, have been used until now for nano- or micro-fibrous mats preparation by electrospinning [57,58,63]. Virgin polyamide has already been electrospun for many applications, such as water or air filtration, composites with antioxidant activity, or improved mechanical properties [53,54,55,56]. HFIP is an acidic alcohol, and due to its strong hydrogen bonding properties, it can be used as a solvent for many different polymers. HFIP has been used for the electrospinning of low-soluble synthetic or natural polymers, such as poly (ethylene terephthalate) (PET) or silk [21,36]. The authors’ experience shows that it is impossible to electrospin from acidic solutions due to the use of user-made (non-commercial) equipment and the inability to control environmental parameters, such as humidity, temperature, pressure, or airflow, in such conditions. HFIP is an expensive solvent that is considered toxic; however, these disadvantages can be partially reduced by mixing with another miscible material, such as chloroform or dichloromethane with a low concentration of HFIP, as it was already used in the chromatographic characterization of PET, poly(butylene terephthalate) (PBT), or polyamides (PAs) [64,65]. Although this solvent was electrospinnable, the resulting polyamide-6 fibers were observed to be thicker but uniform [66,67,68,69].

On the other hand, dichloromethane is widely used in the pharmaceutical industry as a process solvent. The residue tolerances were established by the Food and Drug Administration (FDA) [70]. In this study, the wasted polyamide was electrospun from the HFIP/DCM solvents mixture in the volume ratio 2:8.

### 3.1. Morphology of Electrospun Membranes

The neat rPA and rPA solutions with various concentrations of MAG were electrospun to obtain the fibrous membranes. To ensure that rPA did not undergo degradation during the electrospinning process, the molar mass and molar mass dispersity of neat rPA membrane were assessed by GPC in the same way as the input material, as described in Section 2.1. The molar mass was M_w_ = 69,100 g.mol^−1^ and molar mass dispersity Ð_M_ *=* 2.08. Compared to the M_w_ and Ð_M_ of input PA, there is no significant change in the molar mass and molar mass dispersity. In this case of first-stage material recycling, the electrospun wasted polyamide did not significantly degrade during the recycling process. In addition, based on general knowledge of waste plastic recycling and material regeneration, it must be said that repeated recycling could cause degradation, which can result in a reduction in molar mass and loss of physical properties [71].

**Figure 1:**
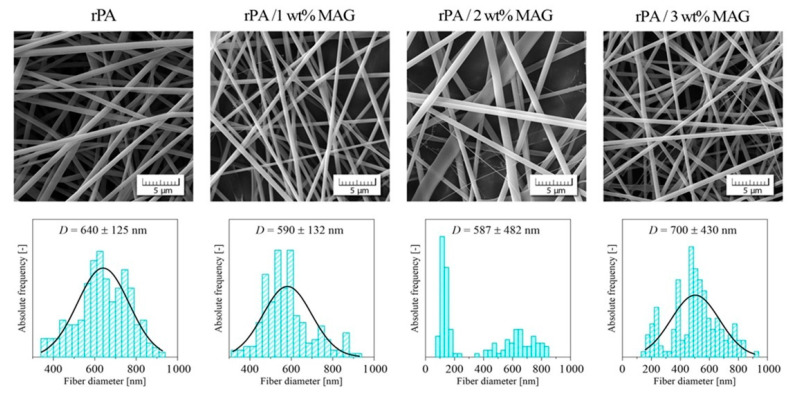
SEM images of the neat rPA and rPA/MAG fibers and fiber diameter distribution plots. Electrospinning parameters were kept constant during the preparation of all four samples. The working tip-to-collector distance was 12 cm. The applied voltage was 20 kV with positive polarity. The feeding rate was 0.2 mL·h^−1^.

The morphologies of the neat and loaded rPA fibrous membranes are shown in Figure 1. It can be observed that the neat rPA membrane exhibits spherical bead-free fibers in the area inspected by SEM. The increasing concentration of MAG had an unexpected effect on the spherical morphology of fibers. As shown from rPA with 2 wt% MAG, the fibers have flat cross sections and high sub-structures containing very thin fibers, forming the continuous web visible in the membrane. Such sub-structures were occasionally produced in the case of high viscosity PA6 solutions, observed by other authors [30]. All selected MAG concentrations significantly exceeded the critical micelle concentration. Therefore, the presence of free micelles in the solution was presumed. It is known that their amount and size increase with increasing the surfactant concentration, which could lead to phase separation.

**Figure 2:**
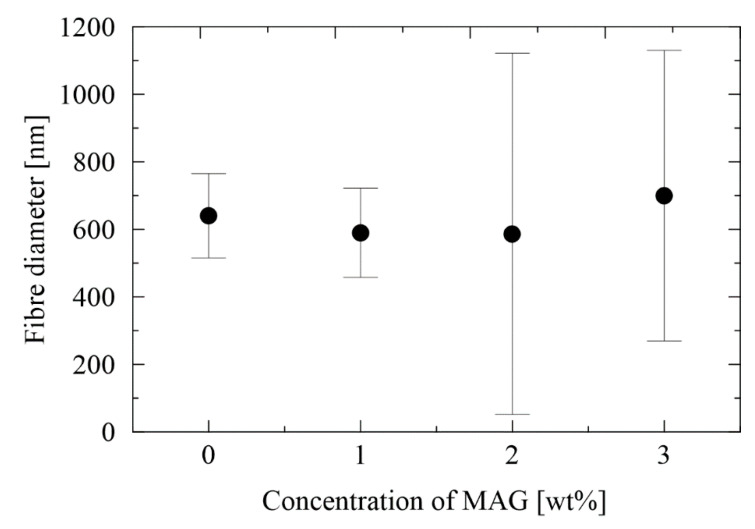
The fiber diameter of the neat rPA and rPA/MAG fibrous layers.

Additionally, given the fiber diameter in Figure 2, the average diameter of a neat rPA sample is 640 ± 125 nm. When the addition amount of MAG increases, the diameter slightly decreases, while the distribution of fiber diameters increases continuously. However, with a higher concentration (3 wt%), the fiber diameter rises again. Non-uniform diameter distribution is obtained with increasing content of MAG, and one option for non-uniform fibers is a change in viscosity. However, the results here show that the nanofiber characteristics cannot be attributed to a single composition parameter.

As shown in Table 3, the estimated pore size and pore size distribution of the rPA membrane was smaller than that of fibrous composites rPA/MAG and showed an increasing trend with the rising MAG concentration. The pore size distribution graphs are shown in Figure S3 in Supplementary Materials.

In filtration, the pore size distribution determines the selectivity and separation efficiency of the membrane as well as the pressure drop across the filter. The pore size may also significantly affect air and water vapor permeability [72]. This effect is discussed in Section 3.5, Assessment of the filtration properties of electrospun rPA and rPA/MAG 3 wt% membranes.

Then with the further increase in the MAG concentration, the average diameter grows again, and fiber diameter distribution is increased. Very thin fibers are also present in rPA with 3 wt% of MAG. One option for non-uniform fibers is a change in viscosity. The second hypothesis can be that all selected MAG concentrations significantly exceeded the critical micelle concentration. Therefore, the presence of free micelles in the solution is presumed. It is known that their amount and size increase with increasing surfactant concentration, which could lead to phase separation.

On the other hand, when the concentration of amphiphilic substances reaches the critical value, the size and amount of the aggregates stabilize [31]. Comparing all selected MAG concentrations, it seems that 2 wt% MAG can be regarded as the critical concentration promoting the phase separation and more specific distribution of fibers. A weaker surfactant polymer interaction can be expected here. This phenomenon decreases with higher monoacylglycerol content (3 wt%). A different effect of various types of surfactants, depending on their structure and ionic character, on PVDF fiber size and morphology was confirmed by Zheng et al. [73]. The least beaded structure was obtained with cationic hexadecyl trimethyl ammonium bromide, while nonionic Triton X-100 led to the variable fiber structure with beads forming according to the specific concentration.

On the other hand, all prepared membranes modified with nonionic monoacylglycerol exhibited a bead-free structure. It is also worth mentioning that this type of surfactant has an excellent environmental profile compared to other surface-active compounds commonly used to incorporate polymer fibers. Triton types are additionally known for risks when an entire degradability process is not performed. The results here show that the nanofiber characteristics cannot be attributed to a single composition parameter.

### 3.2. ATR-FTIR Analysis

**Figure 3:**
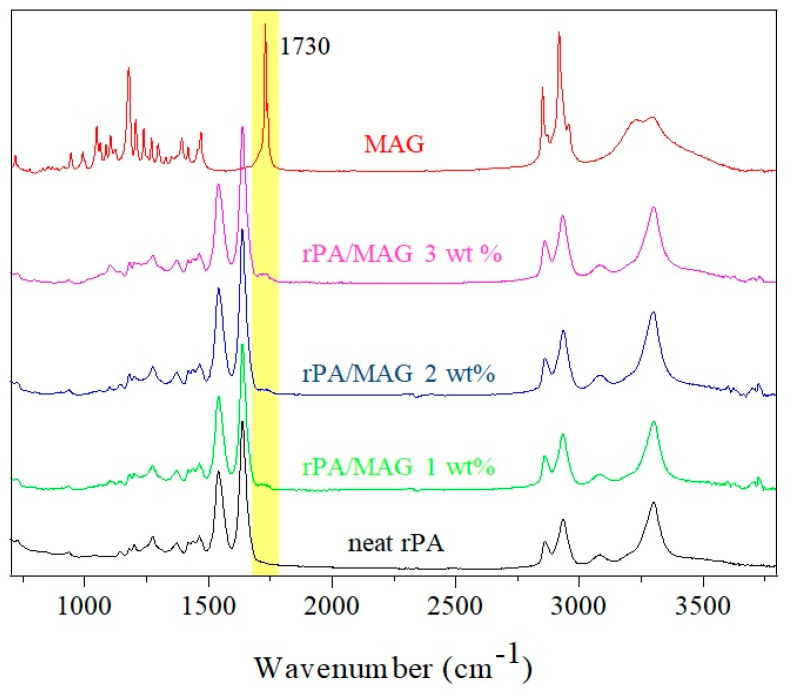
ATR-FTIR spectrum of neat rPA, MAG, and rPA/MAG composites.

ATR-FTIR spectra of the electrospun neat rPA and rPA/MAG composites with various concentrations of MAG are shown in Figure 3.

The typical characteristic bands of polyamide 6 are observed in the spectra rPA, rPA/MAG 1 wt%, rPA/MAG 2 wt%, and rPA/MAG 3 wt%. In these spectra, the band at 1540 cm^−1^ is assigned to deformation vibrations of N–H, and the band at 1630 cm^−1^ corresponds to C = O stretching. The peaks at 2855 and 2925 cm^−1^ are present due to C–H stretching vibrations in alkyl groups. The peak nearby 3300 cm^−1^ is associated with an absorption band of the hydroxyl group.

Spectra of MAG showed peaks at 2950 cm^−1^, 2850 cm^−1^, and 1730 cm^−1^, corresponding to carboxylic acid bands. In rPA electrospun fibers enriched with 3 wt% MAG, the characteristic peak at 1730 cm^−1^ is the most significant. It is assumed that with the decreasing concentration of MAG (1 and 2 wt%), the edge of the detection limit is reached. Therefore, the peak indicating the presence of MAG is insignificant (see a yellow area in Figure 3).

Based on ATR-FTIR analysis, it can be concluded that the base PA polymer structure is maintained after MAG addition. Therefore, no substantial effect on the constituents’ interaction is expected.

### 3.3. Thermogravimetric Analysis and First-Order Derivatives (TGA)

**Figure 4:**
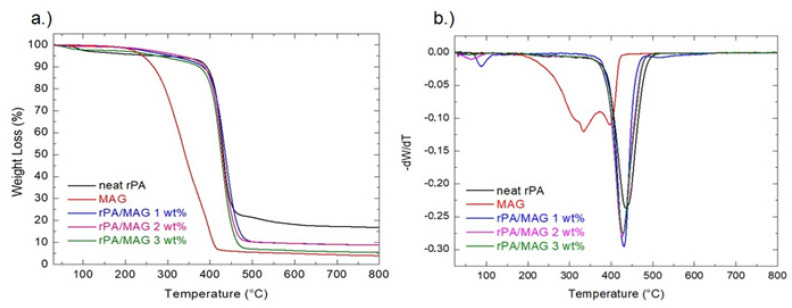
TGA thermograms for electrospun neat rPA, MAG and composites: rPA/MAG 1 wt%, rPA/MAG 2 wt%, rPA/MAG 3 wt% (**a**). Graphs of 1st derivation of TGA (**b**).

The TGA was used to estimate the thermal stability and compatibility between the two components of the investigated samples. The results are shown in Figure 4.

The thermogram of MAG shows a weight loss of approximately 96% at 800 °C and, at least, two thermal events between 160 and 430 °C. These thermal events can be observed by the slopes in the curve. The decomposition of MAG started at 160 °C. The 1st order derivative, which refers to the temperature where maximum mass decomposition occurred, revealed the two maxima at around 330 °C and 390 °C.

The TGA curve of rPA shows the thermal stability up to the decomposition temperature at about 435 °C. The first weight loss of about 5% of rPA is observed close to 100 °C due to moisture loss due to the hygroscopic nature of the polymer. The total weight loss at 800 °C is about to 86%. The 1st order derivative confirmed that the temperature of decomposition of PA is 435 °C. The decomposition temperature of the samples rPA/MAG 1 wt%, rPA/MAG 2 wt%, and rPA/MAG 3 wt% was 430 °C, 428 °C, and 426 °C, respectively. The decomposition temperature changed only very slightly due to the small concentration of MAG. However, lowering the decomposition temperature and the presence of only one melting peak can be considered a sign of good compatibility of the two components.

### 3.4. Wettability of Electrospun Membranes

**Figure 5:**
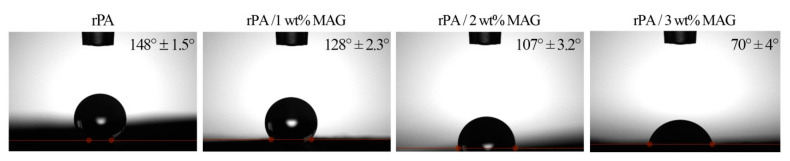
The water contact angle of the neat rPA and rPA/MAG fibrous membranes after 4 s.

To compare the surface wettability of fibrous layers, water contact angle measurements were carried out. The water contact angle (WCA) of rPA is 148°, indicating the hydrophobic surface. The drop in contact angle associated with enhanced surface hydrophilicity occurred with the increasing concentration of MAG in rPA fibrous layers; see Figure 5.

**Figure 6:**
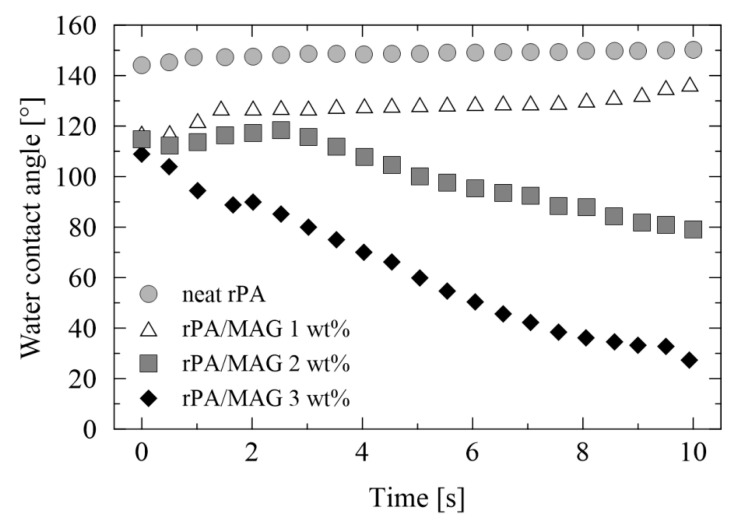
The water contact angle of fibrous membranes in dependence on time.

The effect of rPA modification was also proved in dynamic WCA measurement in time. As illustrated in Figure 6, the WCA of neat rPA is almost constant in a given time interval (10 s), while the modified rPA fibrous membranes (2 and 3 wt% of MAG 12) exhibit a drop of WCA with time. This is due to the character of MAG and the type of its adsorption with hydrophilic groups oriented into the surrounding. In the previous studies, a similar behavior at PVB/MAG 10 and PVDF/MAG 12 fibrous membranes was found [74,75].

### 3.5. Assessment of the Filtration Properties of Electrospun rPA and rPA/MAG 3 wt% Membranes

The filtration efficiency (E) was measured on self-supporting membranes electrospun from neat rPA and rPA/MAG 3 wt% on at least three samples from each investigated membrane according to the requirements of the standard EN 1822 applied to high- and very high-efficiency air filters with ultra-low penetration (EPA, HEPA, and ULPA) used in the field of ventilation and air conditioning, as well as in technological processes, such as clean-room technology or the pharmaceutical industry [76]. The studied membranes could not be classified according to the standards EN 143 or EN 149 intended to classify filter surface material or half-mask and respirators, respectively, due to the too high pressure drop (ΔP) (Table 4). To compare with the available literature, that should be under 100 Pa due to the user comfort [36]. The thicknesses of the membranes were 0.07 ± 0.001 and 0.08 ± 0.02 mm, respectively. These samples were selected to investigate the possible impact of MAG and pore size on the filtration effectivity and air and water vapor permeability. All the measured data, including the standard deviation, are listed in Table 4.

**Figure 7:**
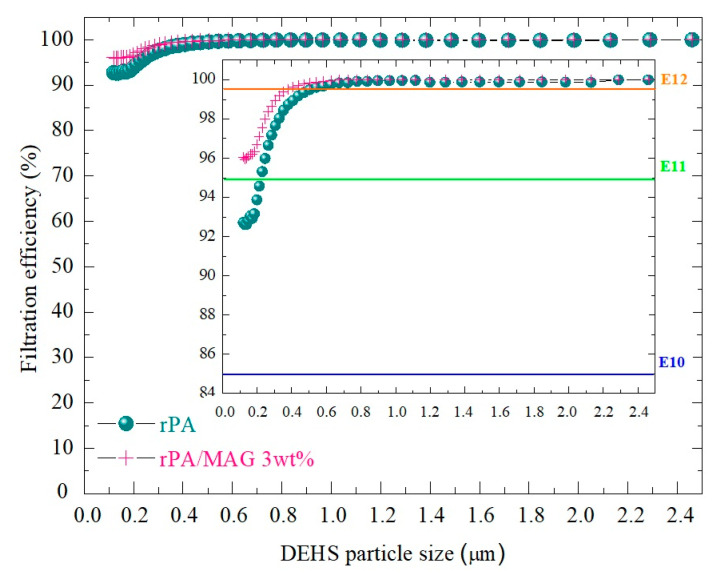
Filtration efficiency (%) of the neat rPA and rPA/MAG 3 wt% membranes. The limits of filtration efficiency for classification into the individual class are indicated here. The figure shows the representative results of one of the three tested membranes from each investigated membrane type.

Recycled-PA electrospun membrane could be classified as E10 (efficiency particulate air filters (EPA) ≥ 85% collection efficiency, penetration <15%), and rPA/MAG 3 wt% as E11 (EPA ≥ 95% collection efficiency, penetration <5%) according to the measured efficiency of the most penetrated particles (Figure 7). The tested samples correspond to higher filtration classes, where prefilters are used to extend the service life. Such filters are compelling enough to protect against germs, bacteria, or metallic-oxide smoke [36,76]. To compare the results in this study, the list of some electrospun polymers and achieved results available in the literature is provided in Table 5.

Although electrospun membranes are inherently favorable for enhancing permeability due to their porous structure, the membrane thickness causes a considerable resistance against air and vapor movement across membranes, resulting in low flux [78]. In agreement with this statement, the pressure drop increased rapidly as the basis weight increased in this study, due to the effects of increasing amounts of fibers in the membrane. It is shown that the filtration efficiency depends on the basis weight. Filtration efficiency increases with the basis weight and thickness increasing.

On the other hand, judging the relative overall performance, the quality factor does not depend on the basis weight, and it is the biggest for the rPA. The larger Q*_f_* indicates a more effective filtration performance, but not for very efficient filters because the efficiency growth over 90% is usually lower than the pressure drop growth. Therefore, the higher effectivity in rPA/MAG 3 wt% is more authoritative. The fiber diameters could indicate better filtration efficiency in the membrane with larger fiber diameter. This result does not necessarily mean that the filtration efficiency depends on the fiber diameter. This can be explained by the fact that the pores’ size distribution and their shape through the membrane with greater thickness and basis weight are not uniform. Fibers with a wide size distribution are randomly deposited in the membrane, which also plays a role.

In the EN 1822 Standard, according to which the tests were performed, cyclic tests are not defined. Therefore, the cyclic performance was not estimated. Of course, it is necessary to expect changes in filtration properties due to filter clogging over time. The increasing pressure drop evidences the clogging of the filter. The pressure drop change was observed during the test procedure, and this value’s growth corresponds with commercial HEPA filters.

Air and water vapor permeability may be increased with large pore size, which can be effective as long as membrane hydrophobicity is high enough to retard pore wetting efficiently [78].

Herein, with increasing surface wettability caused by the increasing MAG concentration, the water vapor permeability increased as well, despite the thickness slightly increasing. This behavior was observed and could be explained by increasing the plasticizer concentration. In this case, the MAG acts as a plasticizer with its amphiphilic structure. An increase in its concentration causes an enhancement in the WVP of hydrophilic membranes due to the increased driving force of the mass transfer.

The air permeability is not affected by the rising of MAG concentration and pore size or its distribution. The thickness and basis weight has no significant impact on the air and water permeability.

### 3.6. Antibacterial Activity of Electrospun Membrane

Antibacterial activity of rPA and rPA fibrous layers loaded with three different concentrations of MAG were tested against Gram-negative and Gram-positive bacteria by the disc diffusion method. Almost all samples showed no inhibition zone against *E. coli* and *S. aureus*, except rPA/3 wt% MAG, exerting a very narrow zone around the sample disk against *S. aureus*. These results correspond with the ATR-FTIR method, where MAG presence was undoubtedly proved. Nevertheless, bacteria were not able to grow under the samples. It can be supposed that the concentration of MAG released from fibers is too low to exhibit greater antibacterial activity. It is known that MAG (monolaurin) is more active against *S. aureus* than *E. coli* [79].

**Figure 8:**
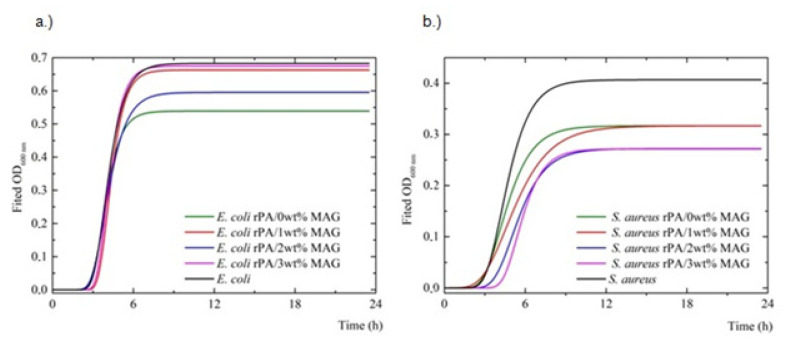
Growth kinetics of bacterial species: (**a**) *E.coli*; (**b**) *S. aureus* alone and with rPA/0, 1, 2, 3 wt% MAG. Lines represent fitted model according to the Gompertz equation.

The growth inhibitory kinetics of bacterial species (*Escherichia coli*, *Staphylococcus aureus*) was investigated by Tecan microplate reader, and optical density (OD) values were measured concerning the time at 600 nm. The experimental data comparing the growth of bacterial samples were plotted and presented in growth curves corresponding to the OD_600_ MAG samples in different concentrations over time, analyzed by the Gompertz method, Levenberg–Marquardt algorithm (Figure 8).

The bacterial growth curve has several typical phases. The initial lag phase consists of limited growth as bacteria acclimate to their new environment. The next exponential phase is where bacteria are most rapidly multiplying. The third stationary phase is characterized by the equilibrium between dividing and dying cells as nutrients are depleted and waste products accumulate. The final death phase is where bacteria start to die as toxic products accumulate [80]. The growth curve of *S. aureus* and *E. coli* bacteria cultivated with rPA and rPA/MAG fibrous layers are illustrated in Figure 8. The population growth kinetics concisely described by growth parameters (λ, μ_max_, and A) was calculated using the Gompertz model (Table 6). Generally, low λ values indicate that bacterial strain can rapidly grow. Antibacterial activity can be noticed by increasing the λ value. High μ_max_ values stay for rapid utilization of substrate by bacteria. High A values, comparable with untreated control growth after 24 h, convince of the low antibacterial effect of the tested treatment [81]. Analysis of the observed data suggests (Figure 8) that the lag phase for *E. coli* lasts for 3.3 h (control growth), and the addition of rPA/MAG did not prolong the lag phase. Similarly, both other parameters (μ_max_ and A) did not significantly differ from the control.

On the other hand, the significantly prolonged lag phase (λ ≥ 1.1 h) can be noticed in *S. aureus* with rPA/2 wt% or 3 wt% MAG (Table 5). Additionally, the maximum specific growth rate is significantly decreased compared to control *S. aureus* growth. Asymptotic values correspond with other parameters. The results reveal a good antibacterial activity for the rPA/2 and 3 wt% MAG fibers against *S. aureus*. In line with the results mentioned above, the reduction effect of fibrous membranes against planktonic bacteria *S. aureus* was proved.

**Figure 9:**
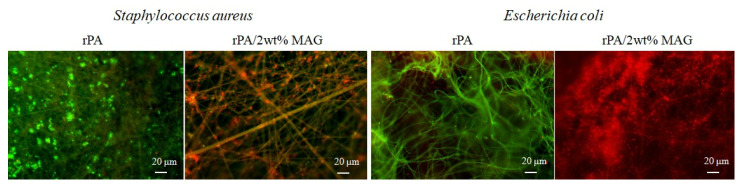
Fluorescence microscopy of LIVE/DEAD bacterial viability assay.

The biofilm on the neat rPA and rPA/MAG layers was observed using two independent experiments. First, the LIVE/DEAD bacterial viability assays were carried out by fluorescence microscopy; see Figure 9. In this image, the live bacterial cells are green, and conversely, the dead bacterial cells are red. It seems that a huge difference was found between neat rPA and rPA loaded with MAG. There were observed no live cells (green) of both *S. aureus* and *E. coli* on the surface of rPA/2 wt% MAG fibrous membrane, which can be explained not only by the presence of MAG, but also by the specific surface electrokinetic properties and hydrophobic interactions as was described earlier [74].

**Figure 10:**
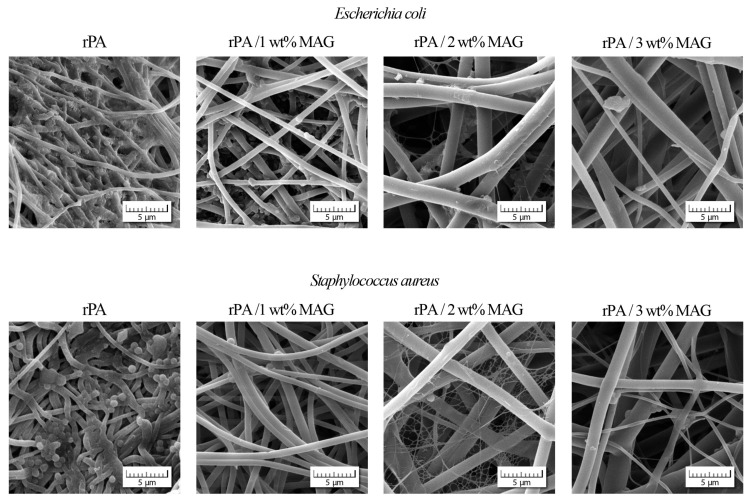
SEM analysis of *E. coli* or *S. aureus* bacteria on the fiber surface after 72 h cultivation.

Further, the SEM analysis was conducted on the fibrous samples (see Figure 10). These results indicate that *E. coli* and *S. aureus* could form biofilm in the case of a neat rPA fibrous layer. In contrast, the rPA fibers with MAG exhibited no biofilm formation after 72 h of bacterial growth. Only a few bacterial cells could be observed and, probably following the fluorescence microscopy results, were supposed to be dead.

A molecule of MAG 12 actually interacts with cell wall surfaces, thus, enhancing the linkage with negative bacterial charge. The antibiofilm effect is probably closely associated with significant changes in the hydrophobic character of fibers and surface charge of applied bacteria (*S. aureus* and *E. coli*). As regards staphylococci, the initial interaction between cells and surface is facilitated by the lipoteichoic acid’s lipid part, which enables bacteria to overcome the natural surface electrostatic resistance [82,83].

## 4. Conclusions

In this work, the electrospun recycled polyamide and polyamide enriched by monoacylglycerol (rPA/MAG) at three different concentrations self-supporting membrane were fabricated. As a solvent, the mixture of HFIP/DCM in the volume ratio 2:8 was used. The antibacterial activity of the prepared samples was investigated.

The produced rPA/MAG fibrous membranes did not reveal any inhibition effect against *E. coli*. However, a demonstrable slowdown of *S. aureus* growth was shown by the prolonged lag phase in the case of the fibers with 2 and 3 wt% MAG. Based on the obtained data, prepared rPA/MAG fibrous membranes can be evaluated as antifouling against both tested bacterial strains and antimicrobial against *S. aureus*.

The filtration efficiency of the two selected membranes was investigated as well. Filtration efficiency of rPA and rPA/MAG 3 wt% was 92.7 ± 1.3% and 96.1 ± 1.7%, respectively. Based on the measured filtration efficiency, the membranes were classified into classes E10 and E11, respectively, according to Standard EN1822. Such membranes could be used as efficient air filters in ventilation and air-conditioning and in technological processes, such as clean-room technology or the pharmaceutical industry. Although the fibers and pore size increased with the addition of MAG, the air permeability of the rPA/MAG 3 wt% membrane was not significantly affected. The water vapor permeability, as well as wettability, was enhanced with the increase in MAG concentration.

The filtration effectivity testing revealed that these membranes based on poly-amide waste and surfactant, which has an excellent environmental profile compared to other surface-active compounds commonly used to incorporate polymer fibers, are not suitable for personal protection facial masks due to the high pressure drop. However, antibacterial and antifouling nanofibrous membranes with high filtration efficiency were produced, ideal for air conditioning and technological processes, such as clean-room technology or the pharmaceutical industry.

## Figures and Tables

**Table 1:** Classification of textile recycling and recycling methods, products, and drawbacks.

Textile Recycling	Recycling Methods	Product of Recycling	Drawbacks	Ref.
Fiber recycling	Mechanical methods	Fibers	- Fibers length is reduced compared to the original as an unwanted side effect; length affects the spinnability. - Some dust will be generated.	[7,8,9,10,11,12]
Polymer recycling	Mechanical methods	Fibers	As fiber recycling	[2,8]
	Physical methods	Polymer suitable for reprocessing (in the form of melt or solution)	- Contaminations with impurities and other polymers - Degradation of material.	[6,7,8,13,14,15]
Monomer recycling	Mechanical methods	Fibers	As fiber recycling	[2,8]
	Chemical/Biological methods	Monomers	- Hazardous - Uneconomical	[7,8,12,16,17,18]

**Table 2:** Characteristics of spinning methods used in the recycling and compared to solution and melt electrospinning.

Type of Spinning	Advantages	Disadvantages	Ref.
Melt spinning	i. High production efficiency; ii. Low cost; iii. Easy large scale production; iv. Ability to create fibers with controlled cross-sections.	i. Thermal degradation of the polymers; ii. Impossible to produce the fibers with incorporated nanoparticles (carbon, metal, active compounds) into polymer blends.	[14]
Solution spinning - evaporation of the solvent - coagulation in a suitable liquid	i. It can be used for any polymer; ii. Fiber can attain strength comparable with maximum theoretical strength; iii. The process can be continuous.	i. Production rate is low; ii. More baths are required to remove the solvent completely; iii. High costly production; iv. The formation of exact fiber cross-section is difficult to control because of the inward and outward mass transfer process.	[15]
Electrospinning - Solution - Melt	Solution: i. Low cost; ii. Available for industrial production; iii. Fibrous can be deposited onto a variety of substrates; iv. Used more than 200 polymers; v. Functionalization of the fibers before, during, and after spinning.	i. Some polymers do not have the appropriate solvent; ii. Evaporation of solvent changes the fiber surface; iii. The used solvent is often toxic; iv. The capillary can be blocked; v. Low productivity of conventional needle electrospinning.	[21,24,25,26,27]
	Melt: i. More environmentally friendly than solution electrospinning ii. Does not use any harmful or toxic solvents	Difficulties in melting the polymers	[28,29]

**Table 3:** The pore size of neat rPA and rPA/MAG fibrous membranes.

The Concentration of MAG [wt%]	MIN Pore Size [nm]	Average Pore Size [nm]	MAX Pore Size [nm]
0	192	840 ± 350	2185
1	365	1130 ± 600	3225
2	345	1190 ± 820	5350
3	530	1740 ± 1070	5540

**Table 4:** Analysis of investigated samples rPA and rPA/MAG 3 wt% for filtration application.

Sample	Basis Weight (g.m^−2^)	E_100nm_ (%)	E_300nm_ (%)	E_600nm_ (%)	Q*_f_* (Pa^−1^)	ΔP (Pa)	B (L.mm.s^−1^)	RWVP (%)	Filter Class *
**rPA**	8.7 ± 0.02	92.7 ± 1.7	97.67 ± 2.0	98.49 ± 1.9	0.035 ± 0.001	129 ± 9	78.6 ± 3.5	95.5 ± 1.1	E10
**rPA/MAG 3 wt%**	9.5 ± 0.02	96.1 ± 3.4	98.18 ± 1.8	99.87 ± 1.8	0.024 ± 0.001	189 ± 12	78.4 ± 3.8	98.2 ± 1.3	E11

* Filter class according to the EN1822.

**Table 5:** Filtration performance of different electrospun polymers.

Sample	Fiber Diameter (nm)	Basis Weight (g.m^−2^)	PM Size (nm)	E (%)	Q*_f_* (Pa^−1^)	ΔP (Pa)	Ref.
Poly(lactic acid) (PLA)	150–300	5.18	260	99.97	0.065	165	[37]
Polyacrilonitrile (PAN)	200	NA	≤100	96.12	0.024	133	[32]
Polyurethane (PU)	120	0.4–0.9	20–400	99.66	0.059–0.029	96–190	[77]
Polyamide 6.6 (PA)	60	0.46	300	90.9	0.034	69	[63]
Poly(ε-caprolactone) (PCL)	922	NA	300–1000	90–97	0.010–0.020	72–510	[34]
Polyvinylalcohol (PVA)	150	NA	≤1000	89.07	0.001	220	[38]
PVA	213–430	16.6–67.6	12–480	≥97	NA	195–2693	[39]
Poly(methyl methacrylate) (PMMA)/polydimethylsiloxane (PDMS)	300–1000	NA	2500	98	NA	21	[41]
Polystyrene (PS)	272–937	12.22	300	99.99	0.065	50–350	[33]
Poly(ethylene terephthalate) (PET)	95	14	120	98.28	NA	NA	[21]
PET	230	12	120	99.97	0.019	414	[36]
PET/silk	127	2.08	120	93.38	0.030	92	[36]
Cellulose acetate (CA)	175–890	NA	4–240	14.80–99.80	max. 0.14	NA	[35]

**Table 6:** Modified Gompertz equation fitting results for *S. aureus* and *E. coli* growth treated by rPA and rPA/MAG fibrous membranes; μ_max_ is the maximum specific growth rate (log CFU.L^−1^.h^−1^); λ is the lag phase (h); A is the asymptote defined as the maximum theoretical achieved value of a relative number of microorganisms (log CFU.L^−1^).

Bacteria	Fibers	λ(h)	μ_max_	A	Adj. R^2^
*E. coli*	-	3.2705 ± 0.0166 ^a^	1.0022 ± 0.0013 ^a^	0.6912 ± 0.0043 ^a^	0.99859
*E. coli*	rPA/0 wt%MAG	3.2541 ± 0.0249 ^a^	0.9904 ± 0.0018 ^b^	0.6574 ± 0.0055 ^a^	0.99707
*E. coli*	rPA/1 wt%MAG	3.3277 ± 0.0433 ^b^	0.9923 ± 0.0039 ^b^	0.7005 ± 0.0059 ^b^	0.99724
*E. coli*	rPA/2 wt%MAG	3.3145 ± 0.0310 ^a,c^	0.8573 ± 0.0022 ^b^	0.6520 ± 0.0044 ^b^	0.99645
*E. coli*	rPA/3 wt%MAG	3.4674 ± 0.0551 ^c^	0.9264 ± 0.0051 ^b^	0.6219 ± 0.0039 ^b^	0.98548
*S. aureus*	-	4.2365 ± 0.0334 ^a^	0.4068 ± 0.0015 ^a^	0.4024 ± 0.0029 ^a^	0.99899
*S. aureus*	rPA/0 wt%MAG	4.1653 ± 0.0268^a, b^	0.3272 ± 0.0009 ^b^	0.3168 ± 0.0019 ^b^	0.99615
*S. aureus*	rPA/1 wt%MAG	4.5644 ± 0.0333 ^a, b^	0.3168 ± 0.0017 ^b^	0.3167 ± 0.0012 ^b^	0.99424
*S. aureus*	rPA/2 wt%MAG	5.0106 ± 0.0532 ^c^	0.2722 ± 0.0016 ^b^	0.2722 ± 0.0036 ^c^	0.99364
*S. aureus*	rPA/3 wt%MAG	5.3916 ± 0.0563 ^c^	0.2716 ± 0.0018 ^b^	0.2765 ± 0.0031 ^c^	0.99176

^a, b, c^ Lower-case letters indicate significant differences (*p* < 0.05).

## Data Availability

Not applicable.

## Supplementary Materials

The following supporting information can be downloaded at: https://www.mdpi.com/article/10.3390/nano12010050/s1, Figure S1: The ^1^H NMR (DMSO-*d*_6_, 400 MHz) spectrum of 1-monolaurin; Figure S2: The ^13^C NMR (bottom black line) and DEPT135 (blue top line) spectrum of 1-monolaurin (DMSO-*d*_6_, 101 MHz); Figure S3: Te pore size distribution of produced fibrous membranes.
