# Supplementary material for: Reuse of Textile Waste to Production of the Fibrous Antibacterial Membrane with Filtration Potential
Source: Nanomaterials (Basel). 2021 Dec 24;12(1):50. doi: 10.3390/nano12010050 (PMC8746662; doi:10.3390/nano12010050)
Supplement: Supplementary file 1 [file nanomaterials-12-00050-s001.zip › nanomaterials-1463261-supplementary.pdf]

nanomaterials

Supplementary Materials

Reuse of Textile Waste to Production of the Fibrous Antibacterial Membrane with Filtration Potential

Alena Opálková Šišková [1,2,*], Pavel Pleva [3,*], Jakub Hrůza [4], Jaroslava Frajová [5], Jana Sedlaříková [6], Petra Peer [3], Angela Kleinová [2] and Magda Janalíková [3]

1 Institute of Materials and Machine Mechanics, Slovak Academy of Sciences, Dúbravská cesta 9, 845 13 Bratislava, Slovakia
2 Polymer Institute of Slovak Academy of Sciences, Dúbravská cesta 9, 845 41 Bratislava, Slovakia; angela.kleinova@savba.sk
3 Department of Environmental Protection Engineering, Faculty of Technology, Tomas Bata University in Zlin, Vavreckova 275, 760 01 Zlin, Czech Republic; peer@utb.cz (P.P.); mjanalikova@utb.cz (M.J.)
4 Institute for Nanomaterials, Advanced Technologies and Innovation, Technical University of Liberec, Studentská 1402/2, 461 17 Liberec, Czech Republic; jakubhruza1@seznam.cz
5 Faculty of Arts and Architecture, Technical University of Liberec, Studentská 1402/2, 460 01 Liberec, Czech Republic; jaroslava.frajova@tul.cz
6 Department of Fat, Surfactant and Cosmetics Technology, Faculty of Technology, Tomas Bata University in Zlin, Vavreckova 275, 760 01 Zlin, Czech Republic; sedlarikova@utb.cz
* Correspondence: alena.siskova@savba.sk (A.O.Š.); ppleva@utb.cz (P.P.)

In this supplementary material the following figures are presented:

Figure S1. The ^{1}H NMR (DMSO-d_6, 400 MHz) spectrum of 1-monolaurin.

Figure S2. The ^{13}C NMR (bottom black line) and DEPT135 (blue top line) spectrum of 1-monolaurin (DMSO-d_6, 101 MHz).

Figure S3. The pore size distributions of produced fibrous membranes.

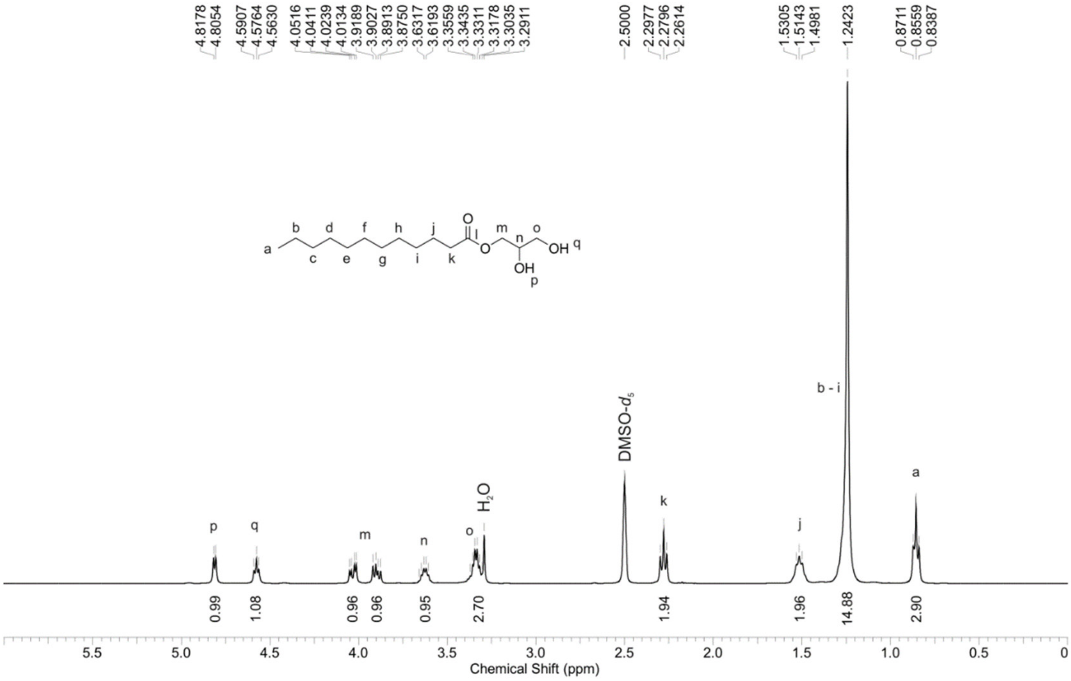

Figure S1. The ^{1}H NMR (DMSO-d_6, 400 MHz) spectrum of 1-monolaurin.

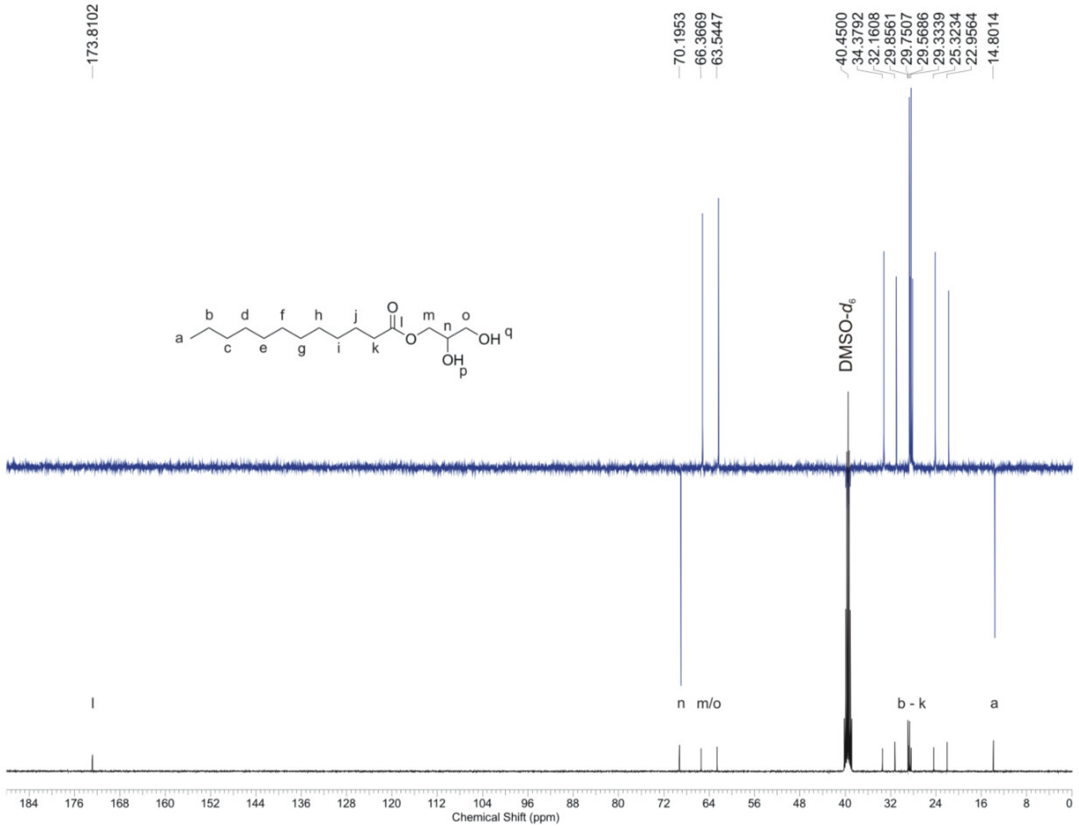

Figure S2. The ^{13}C NMR (bottom black line) and DEPT135 (blue top line) spectrum of 1-monolaurin (DMSO-d_6, 101 MHz).

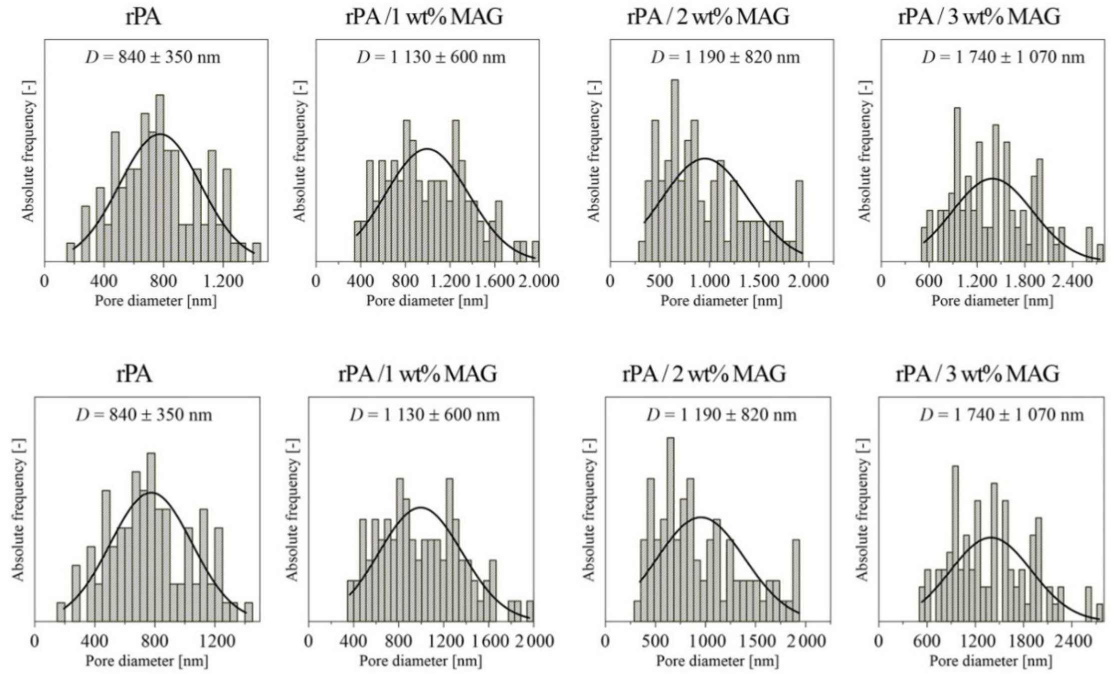

Figure S3. The pore size distributions of produced fibrous membranes.
